# Preventing weight gain: the baseline weight related behaviors and delivery of a randomized controlled intervention in community based women

**DOI:** 10.1186/1471-2458-9-2

**Published:** 2009-01-03

**Authors:** Catherine Lombard, Amanda Deeks, Damien Jolley, Helena J Teede

**Affiliations:** 1 Jean Hailes Foundation for Women's Health Research Unit, School of Public Health and Preventive Medicine, Monash University, Locked Bag 29, Monash Medical Centre, Melbourne, 3168, Australia; 2 Monash Institute of Health Services Research, School of Public Health and Preventive Medicine, Monash University, Locked Bag 29, Monash Medical Centre, Melbourne, 3168, Australia

## Abstract

**Background:**

Women aged 25–45 years represent a high risk group for weight gain and those with children are at increased risk because of weight gain associated with pregnancy and subsequent lifestyle change. Average self-reported weight gain is approximately 0.60 kg per year, and weight gain is associated with increased risk of chronic disease. There are barriers to reaching, engaging and delivering lifestyle interventions to prevent weight gain in this population.

**Methods:**

This study investigated the baseline weight related behaviors and feasibility of recruiting and delivering a low intensity self-management lifestyle intervention to community based women with children in order to prevent weight gain, compared to standard education. The recruitment and delivery of the cluster-randomized controlled intervention was in conjunction with 12 primary (elementary) schools. Baseline data collection included demographic, anthropometric, behavioral and biological measures.

**Results:**

Two hundred and fifty community based women were randomized as clusters to intervention (n = 127) or control (n = 123). Mean age was 40.4 years (SD 4.7) and mean BMI 27.8 kg/m^2 ^(SD 5.6). All components of this intervention were successfully delivered and retention rates were excellent, 97% at 4 months.

Nearly all women (90%) reported being dissatisfied with their weight and 72% attempted to self-manage their weight. Women were more confident of changing their diet (mean score 3.2) than physical activity (mean score 2.7). This population perceived they were engaging in prevention behaviors, with 71% reporting actively trying to prevent weight gain, yet they consumed a mean of 68 g fat/day (SD30 g) and 27 g saturated fat/day (SD12 g) representing 32% and 13% of energy respectively. The women had a high rate of dyslipidemia (33%) and engaged in an average of 9187 steps/day (SD 3671).

**Conclusion:**

Delivery of this low intensity intervention to a broad cross-section of community based women with children is feasible. Women with children are engaging in lifestyle behaviours which do not confer adequate health benefits. They appear to be motivated to attend prevention programs by their interest in weight management. Interventions are required to strengthen and sustain current attempts at achieving healthy lifestyle behaviours in women to prevent weight gain.

**Trial Registration Number:**

ACTRN 12608000110381

## Background

Women in the age group 25–45 years represent a high risk group for weight gain in Australian adults. Average self reported weight gain is approximately 0.60 kg per year in this age group.[1] Weight gain in women is associated with significant ill health. An increase in body mass index (BMI), even within the healthy weight range, is associated with an increased risk of diabetes and cardiovascular disease (CVD).[2]

Women with young children are at particular risk because of weight gain associated with pregnancy [3] and low levels of physical activity.[4] These women are a vital target for the promotion of healthy lifestyles, as they make many of the daily food and activity decisions for families, influencing children, and partners eating and physical activity patterns. [5] Prevention of weight gain targeting women with children has potential to achieve significant health benefits for both women and their families.

Interventions to prevent weight gain to date have been inadequate and generally ineffective.[6,7] High intensity interventions including frequent contact with facilitators and personal ongoing support achieve weight loss but are not considered feasible in large populations.[8] Lower intensity interventions hold the most potential in their ability to reach a broad population at lower cost, but have had mixed success.[9,10] Lemmens et al recently reviewed interventions for the prevention and treatment of obesity, but not all of the studies were intentionally designed to prevent weight gain. The authors included interventions which reported the effect of interventions on weight or body composition as secondary outcomes. Interventions designed specifically to prevent weight gain will be distinctly different from those aiming for weight loss or single component behavior change. Direct comparison between interventions to change diet or physical activity with those to specifically prevent weight gain fails to identify success factors, the necessary intensity, cost and address gaps in evidence.

Criticisms of lifestyle interventions have included a reliance on self reported data, a lack of control groups, the short term nature and lack of a clear theoretical model of behavior change. In addition, they frequently target specific disease states or recruit motivated volunteers. They have also failed to address, or report on, known barriers to participation in lifestyle related activities cited by women. These include such factors as the number or age of children, a lack of motivation, lack of time, child care difficulties, social support, cost, travel and convenience.[11] Reports on lifestyle related behaviours in women rarely stratify for number or age of children and consequently we know little about the health related behaviours specific to women with children. Large scale, low-intensity, local, community-based, interventions are required to reach and engage women and demonstrate feasibility of delivery and effectiveness in the prevention of weight gain.

The Healthy Lifestyle Program (HeLP) incorporated successful strategies from relevant research into a low intensity lifestyle intervention. We included, social support, goal setting, self-monitoring, relapse prevention training. [12,13] We measured self-efficacy for specific health related behaviors and stage-of-change. Previous research has reported programs that are personalized with some face to face contact, and include on-going support, provide improved outcomes.[14] There is also evidence of short term success in interventions aimed at people connected at the local community through church groups.[15] We therefore developed a multi-factorial intervention that builds on existing social support networks and includes personal delivery and on-going support.

The present study reports on the recruitment of a broad cross-section of women with children into a low intensity lifestyle intervention. We describe the intervention, the delivery of phase 1, (the face to face component) of the one year intervention, and report on the health related behaviours in this community based population of women.

## Methods

HeLP is a cluster-randomized, controlled, lifestyle intervention. The target population is community-dwelling, generally healthy women who are mothers of at least one child (aged 5–13 years) attending a primary school. The setting for the recruitment and delivery of the intervention was primary schools in one local government area (total population 130,000), of moderate socio-economic disadvantage based on the Socio-Economic Index for Areas (SEIFA), created from population census data.[16] Body mass index (BMI) was not used as an exclusion or inclusion criteria in order to be inclusive of the all members of the community.

Participants were excluded if taking prescribed weight control medications, breastfeeding of infants under 6 months, were pregnant, became pregnant during the study, wished to gain weight or suffered with a serious physical or psychological illness that would prevent taking part in the study. We obtained approval from the Southern Health Human Research Ethics Committee (ref 05187C) and informed consent was obtained in writing from all participants.

### Recruitment and randomization strategy

We identified all primary schools within this geographical area (n = 22) and invited schools to participate progressively until the desired number agreed (n = 12). One school refused to participate, reporting they participate in many research projects; one did not have enough space to accommodate the program. The schools were then paired with a school of equal size, and each pair of schools was randomly allocated to intervention (n = 6) or control (n = 6) using computer generated numbers. Schools were paired by size to give a relatively equal denominator for recruitment in both groups. The study sampling and intervention was designed to target clusters of mothers associated with particular schools, to reduce possible contamination between participants.

**Figure 1:**
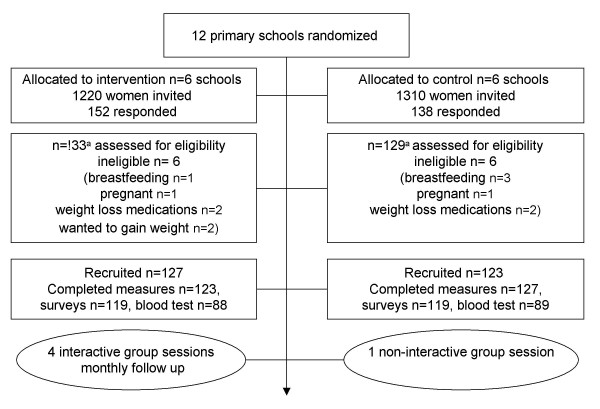
**Flow chart of subject enrolment, random assignment and completion of the intervention (4 group behavioral sessions)**. ^a ^n = 28 responded initially but were unable to attend at the allocated times.

We invited all mothers within the primary schools to participate by letter, then register interest by return mail, phone or fax. The women were given information packs containing an explanation of the study, questionnaires, plus a sealed research pedometer, with instructions on how to wear the pedometer. Recruitment and delivery occurred progressively from May 2006 until August 2006 (see figure 1). The group facilitator randomized schools and delivered the program and so was aware of the allocation of participants. Data scoring and entry was completed by research members blinded to group allocation.

### Delivery

#### Control group

The control group attended a single thirty minute, group, non-interactive, health education lecture at the local school their children attended. Content was based on the Australian Dietary Guidelines and the Australian Physical Activity Guidelines and they received readily available pamphlets based on these Guidelines.[17,18] Participants were weighed, measured and completed all baseline questionnaires at this session. Participants were not given results of baseline measurements until after recruitment so as not to influence participation. They received a pedometer to use as they wished over the year, but no daily step goal. They were given a request to have a fasting blood sample taken. They received no further support, but completed a brief mailed questionnaire at 4 months and return for final data collection at 12 months.

#### Intervention Group

The intervention content was based on the social cognitive theory, specifically goal setting, self monitoring, social support, observational learning, problem solving and relapse prevention training, offering multiple avenues to behavior change.[19]

Phase one of the intervention consisted of three interactive group behavior change sessions delivered in the first month by an experienced dietician and a fourth session at four months. Participants were weighed, measured and completed all baseline questionnaires at the first session. Participants were not given results of baseline measurements until after recruitment so as not to influence participation.

Content included evidence based messages with clear goals on diet, (e.g. eat 2 serves fruit and 5 serves vegetables each day), physical activity (e.g. aim for 8–10,000 steps per day) and behavior change (e.g. monitor yourself regularly) and written handouts were provided. During group sessions outcome expectancies were discussed in order to clarify the intervention aim, that is, to prevent weight gain, not promote weight loss. Emphasis was placed on self monitoring through the use of one or more of the following, regular self-weighing, use of a pedometer, or a diary. We discussed and demonstrated goal setting, as well as problem solving and relapse-prevention skills, which were practiced and personalized; for example, working in groups, participants discussed their own goals, identified problems and were provided with individual feedback. Participants then prepared a personal action plan to trial new behaviors and correct behaviors through feedback the following week. Women were encouraged into voluntary school based walking groups or to walk with friends for social support. Participants were weighed and measured and completed baseline questionnaires at the first group session.

The visit at 4 months reinforced lifestyle and behaviour messages and repeated anthropometric data collection and questionnaires in the intervention group.

All sessions were held in groups, ranging in number from 10–30 participants, at the local primary school. Sessions were informal, brief (1 hour) and accommodated young children. Session times were flexible and could be adjusted to meet the needs of each group. For example, one group of working mothers preferred weekend sessions, in another, sessions were held immediately after school hours to assist part time working mothers. Otherwise sessions were held immediately after dropping children at school or immediately before collecting children at the end of the day.

Phase 2 of the intervention is ongoing support for 1 year, after which participants will return for final data collection. This support will be provided by mobile phone text messages; there will be no further personal contact. In this report we describe results from phase 1(face to face component) of the intervention.

### Measurements

#### Anthropometric measures

Weight was measured on an electronic scale to the nearest 0.1 kg (Tanita model BWB-800 digital scale, Wedderburn Scales, Melbourne, Australia) calibrated prior to weighing periods. Weight was measured in light clothing without shoes with an empty bladder, at the same time of the day, not fasting. Height was measured using a portable stadiometer (Mentone Education Centre, Melbourne, Australia). Waist measurement was taken directly on the skin in a relaxed standing position at the end of expiration with arms hanging freely with an inelastic plastic fiber tape measure. The measurement was taken perpendicular to the umbilicus and horizontal to the floor to ensure repeatability of measures. The technique utilized the umbilicus as an anchor. This method provides an acceptable measure of waist circumference, but may overestimate circumference when compared to other measures.[20,21] As repeatability was important and privacy and time was limited it proved to be a convenient method. All tape measurements were performed by a single trained researcher.

#### Biological measures

A commercial pathology provider (Melbourne Pathology) collected and analyzed fasting cholesterol, high density lipoprotein, low density lipoprotein, triglyceride and glucose. If participants did not have the request completed within two weeks they were contacted and reminded, with the aim of having blood collected within one month of baseline. For lipids, blood was collected in SST (plain) 8 mL tube, allowed to clot for 30 mins at room temperature and centrifuged for 10 mins at 1000 × *g*. Blood for glucose was collected in Fluoride Oxalate 10 mg/8 mg, 4 mL tube and centrifuged for 10 mins at 1000 × *g*. Serum lipids and plasma glucose were analyzed using Hitachi Modular Analyzer (Roche Diagnostics, USA).

#### Diet and physical activity measurements

We measured dietary intake using the Cancer Council of Australia, Food Frequency Questionnaire analyzed using NUTTAB95 software (Food Standards Australia and New Zealand). The International Physical Activity Questionnaire short version (IPAQ) measured usual weekly physical activity. Both have been validated in adult populations.[22,23]

Physical activity measured by IPAQ was expressed as a categorical variable by calculating the MET-minutes per week (MET-mins = MET level × minutes per day × days per week) where 1 MET is equivalent to resting energy expenditure. Low activity is described as achieving less than 600 MET-mins per week) moderate at least 600 MET-mins per week and high activity, at least 3000 MET-mins per week).[24]

As an objective measure of physical activity, we instructed participants to wear a research pedometer (Yamax Digiwalker, model SW700, Yamax Corporation, Tokyo, Japan) for a min 3 days and max 7 days prior to the baseline measurements and information sessions. The Yamax, digiwalker has been shown to be reliable and a minimum of three days of measurement has been found to be sufficient to estimate free living adult pedometer determined physical activity.[25,26] We sealed the pedometers to prevent feedback on step counts motivating participants to be more active as has been reported elsewhere.[27] Participants kept a daily diary of hours the pedometer was worn, and were asked to record each time it was removed or forgotten. Faulty pedometers were discarded. A full day was deemed to be wearing the pedometer for at least 8 hours, and a half day, wearing it for less than 8 hours but at least 3 hours. Daily steps were calculated by dividing the total steps with the number of days and half days worn.

#### Psychosocial measurements

The Eating and Exercise Confidence Scale developed by Sallis was adapted to measure self efficacy[28]. The reported reliability for the domains of physical activity and eating are 0.68 and 0.43–0.68 respectively and internal consistency was 0.83–0.85 and 0.85–0.93 respectively. This scale has been closely aligned with six sub-factors for the eating scale and 2 for the physical activity scale. These are resisting relapse, reducing energy, eating low fat foods, increasing consumption of fruit and vegetables. For the physical activity scale the sub-factors are resisting relapse and finding time to exercise. Wording was modified to match Australian foods.[28] Questions related to low salt foods were removed as they were deemed irrelevant to this intervention. An additional question was included "How confident are you that you can control your weight if you wished?" to measure self efficacy for weight control. Self efficacy total and individual factor means were determined from a Likert scale from 1–5.

A previously developed Stage-of-Change Scale was used to assess separate stages for physical activity, diet and preventing weight gain.[29,30] Stage-of-change was scored using the algorithms developed for use with the measurement tools. The Self Management Strategy Questionnaire [31] was previously developed and used in young adults mostly female, validated in adolescents and includes items related to cognitive and behavioral strategies related to physical activity. The wording was adapted for this population e.g. 'posting cues for physical activity' was changed to 'make backup plans to be sure I stay active'. Questions were added on diet related strategies and scored on a Likert scale (1 = never to 5 = always).

### Statistics

The estimated sample size required was calculated based on a 600 g difference in weight between intervention and control participants at 12 months, the average weight gain in young Australian women (600 g SD1100 g). To account for the cluster design of the study, a commonsense estimate of an intra-cluster correlation (ICC) of 0.02 and estimate of 30 women per cluster was applied to estimate the sample size. The aim therefore was to recruit 110 participants for each group (at 90% power with the level of significance p = 0.05) as 5–6 clusters. Data was first plotted and sample means, standard deviations, percentages and proportions calculated for relevant demographic and health characteristics of the intervention and control groups separately. Students t-tests were then used to compare means between groups on all individual demographic and health variables. Questionnaires were scored and mean baseline results are presented for the combined intervention and control groups. We used Stata 9, statistical software program (StataCorp, Texas, USA) for all analyses.

## Results

### Recruiting, Delivery and Participation

Three hundred women responded to the invitation sent to 2530 women. We recruited two hundred and fifty (250) women, who were randomized to intervention (n = 123) and control (n = 127) (see figure 1). In comparison with available population data the recruited sample was representative of the population of Australian women of similar age with children, although fewer full time and more part time workers were recruited (see table 1). We recruited a representative proportion of single mothers (10.7%) and women born overseas (25%). The intervention and control groups were similar in terms of demographic and health characteristics indicating successful randomization (see table 2).

**Table 1:** Participant demographic characteristics

	Control * n = 123	**Intervention** **n = 127**	**Australian Population^a^**	**Local Population^b^**
**Mean Age**	40.5 years (SD 4.5) (range 30–55)	40.3 years (SD 4.5) (range 30–55)	n/a	n/a
**Lone mothers**	%	%	%	%
(with child < 15 years)	10.5	10.8	10.6	8.5
**Employment**	%	%	%	%
(women with a child <13 years)				
Not working	43	41	41	36
Part time	51	53	39	40
Full time	6	6	20	19
**Highest education**	%	%	%	
(Women aged 25–54)				
Min Year 10	29	22	38	-
Year 12	22	21	45	-
Trade or certificate	21	27	n/a	-
University and higher	28	29	17	-
**Born overseas**	%	%	%	%
(Women with a child under 13 yrs)	25	26	18	31
**Household Income AUD^c^**	%	%	%	%
<20	14.2	5	11	10.4
$20–40,000	8.4	12.6	23	21
$40–60,000	15.1	21.8	18.6	19
$60–80,000	21.8	16.8	10.5	11
$80,000 +	27.7	26.0	22.8	24
Did not want to answer	12.5	16.7	n/a	n/a

^a ^Australian Population Census, 2001, females aged 25–54 years, Australian Bureau of Statistics, .

^b ^Local Population is the defined as the local government area from where participants were recruited see Australian Population Census, 2001, Australian Bureau of Statistics.

^c ^AUD, Australian Dollars

n/a = not available

* No significant difference between intervention and control

**Table 2:** Participant health characteristics

	**Control (n = 123**) * mean (SD)	**Intervention(n = 127)** mean (SD)
**Weight**	74.6 kg (16.1)	73.3 kg (13.9) **
**Waist circumference**	96.8 cm (14.6)	94.5 cm (12.8)
**BMI**	28.1 kg/m^2 ^(5.8)	27.4 kg/m^2 ^(5.1) **
<25 kg/m^2^	30%	39%
25–29.9 kg/m^2^	43%	34%
>30 kg/m^2^	27%	27%
**Blood Lipids^a^**	n = 88	n = 89
Total cholesterol mmol/L	5.04 (0.10)	4.80 (0.09)^b^
LDL cholesterol mmol/L	2.81 (0.08)	2.63 (0.08)
HDL cholesterol mmol/L	1.70 (0.04)	1.70 (0.04)
Triglyceride mmol/L	1.13 (0.66)	1.03 (0.72)
**Physical activity^c^**	(n = 114) %	(n = 112) %
Low	46	44
Moderate	39	43
High	15	13
**Diet^d^**	(n = 107)	(n = 111)
Energy kJ/day	6791(2383)	6774 (2465)
Fat g/day	68.1 (29.3)	67.5 (28.2)
Saturated fat g/day	27.7 (12.8)	26.6 (11.9)

^a ^n = 177 completed blood test

^b ^between group difference in total cholesterol p = 0.09

^c ^n = 226 accurately completed the physical activity questionnaire (IPAQ).

^d ^n = 218 accurately completed the Cancer Council Food Frequency Questionnaire.

* No significant difference between groups (p > 0.05) ** p = 0.4

Participants were required to attend in person for at least two of the three intervention group sessions. If a single session was missed the information was personally delivered at an alternate time. Therefore all information intended to be delivered was delivered to all participants. Attendance figures for the intervention group sessions were 98% for session 1, 80% for session 2 and 70% for session 3. The most common barriers to attendance were illness in the participant or their children or a change in work arrangements. One facilitator was able to successfully deliver all components of the intervention.

Eighty five percent (85%) of women in the intervention group found the group sessions to be very helpful or helpful compared to 63% of the education only control group. Strategies found to be valuable by intervention subjects were 'delivery by a health professional', 'the sessions were held at the school', 'the pedometer', 'phone mail and text reminders', 'having someone weigh me' and 'handouts on physical activity and diet' in that order, and the least helpful was 'getting a walking group started at school'. Retention rates were high with 97% remaining in the trial at 4 months.

### Health related characteristics and behaviors

Thirty four percent (34%) of participants were within normal BMI (≤ 24.9 kg/m^2^), 38% were overweight (BMI 25–29.9 kg/m^2^) and 28% obese (BMI ≥ 30 kg/m^2^). Prevalence of any type of dyslipidemia was 33% in this population of women (see table 2).

We estimated average energy intake was 6782 kJ per day and average fat intake 68.1 g per day which is equivalent to 32% of total energy. Thirteen percent (13%) of energy was derived from saturated fat (see table 2).

We obtained 191 complete sets of pedometer data after removing those where, a diary was not completed, the pedometer was worn for less than three days, or was faulty. The average pedometer steps per day were 9135 (SD 3607) in the intervention group (n = 100) and 9240 (SD 3735) steps in the control group (n = 91), and overall 67% of women reported less than 10,000 steps per day. The pedometer measures only steps and is helpful in measuring incidental activity which is difficult to capture in questionnaires. In addition the IPAQ questionnaire asks women to report participation in various intensity activities. In this group of women 45% were categorized as low activity (less than 600 MET-mins/week, 41% moderate activity (at least 600 MET-mins/wk) and 14% high activity (at least 3000 MET-mins/wk). Thirty one percent (31%) reported participation in some type of vigorous activity in the past week. The stage-of-change questionnaire reported similar results, where 40% of women reported they are not active enough and are thinking of becoming active (stage 2, contemplation stage) and another 21% had made some changes (stage 3, action stage).

In order to investigate the modeling of healthy behaviors by mothers for their children, participants were asked what activities were performed with children in the past week. Seventy four percent (74%) reported they had walked with their children in the past 7 days, 40% had gone to the park with their children and 20% had ridden a bike with their children. Ten percent (10%) had done no physical activity with their children in the past 7 days.

Weight management practices were investigated. Ninety percent (90%) of participants would prefer to weigh less, yet only 27% of women had spoken to their General Practitioner (GP) about weight in the past year. Even less women discussed physical activity (18%). Many women were attempting some form of dietary self restriction, as 72% reported they had attempted to limit intake in order to lose weight over the past year. Table 3 reports weight related behaviors according to BMI category.

**Table 3:** Baseline weight related behaviors in all participants distributed by BMI category.

**Weight related behavior**	**BMI <25**	**BMI 25–29.9**	**BMI >30**
**I have attempted to limit how much I ate in order to lose weight during the last years**	n = 80 %	n = 93 %	n = 65 %
Never	45	20	17
1–4 times	25	46	37
5–10 times	10	7	17
More than 10 times	14	20	21
I am always on a diet	6	7	8
**During the past month how dissatisfied have you felt about your weight**			
Not dissatisfied	22.5	0	3.0
A little dissatisfied	41	22.5	4.6
Somewhat dissatisfied	16.25	26.8	12.3
Quite dissatisfied	12.5	28	20.0
Very dissatisfied	7.5	22.5	60
**Total energy (kJ)**	6259	6730	7313
**Average daily pedometer steps**	9833	8906	8929
**Total fat (g)**	65	69	74

The stage-of-change questionnaire reported over half of the participants were classified as stage 3, actively trying to stop from gaining weight (58%). Eighteen percent (18%) were seriously considering stopping weight gain (stage 2, contemplation) and 10% were not thinking about weight (stage 1). Only 13% reported they had successfully maintained their desired weight over the past six months (stage 5, maintenance). This was in contrast to the 24% who claim to have maintained activity (stage 5) and the 28% who claim to have maintained a low fat diet (stage 5), with another 13% reporting they had made recent changes to a low fat diet (stage 4).

Self efficacy was measured on a Likert scale range from 1 'not confident' to 5 'very confident'. Participants were more confident they could change their diet (mean score 3.2) than engage in physical activity (mean score 2.7) and were more confident they could stick to an eating plan (mean score 3.0) than stick to an activity plan (mean score 2.7). The reported use of self-management for physical activity strategies (mean 2.52) was similar to diet strategy use (mean 2.59).

## Discussion

This community based lifestyle intervention is feasible based on the reported population reach, attendance, retention and resources required to deliver the program. Population reach is demonstrated by successfully recruiting a representative sample of the target population, that is, women with young children. We delivered all components of the program and achieved high attendance and retention rates. One trained facilitator was able to deliver all intervention components.

The primary school as a setting for recruitment and delivery was successful, addressing a number of barriers to participation by women in health programs. the school is an existing trusted community resource with strong social connections often formed between parents. This social support has been found to have positive influence on physical activity participation. The school is accessible to most women, often within walking distance, and is familiar. Using the school we successfully attracted working-mothers, single-mothers and women from culturally diverse backgrounds, all income levels and education levels. School principals strongly supported the intervention and actively assisted the recruitment. As recruitment rates at each school varied, characteristics of the parent body may affect participation and warrants further investigation.

Overall approximately 11% of invited participants responded. In the context of population reach this is still a significant proportion. Improving the health of 11% of the population would equate to an important public health campaign. This setting gave us access to all women with children who were unselected in terms of health risks.

As expected, we attracted those who were overweight and obese, yet still attracted many leaner women. This is encouraging for future interventions aiming to prevent rather than treat obesity. Almost all of the participants (90%), reported they would like to weigh less, confirming women are generally dissatisfied with their body weight and shape. More than a third of women wished to weigh 1–5 kg less, suggesting weight gain to date is small in many women and potentially reversible. These women are therefore ideal candidates for interventions to prevent obesity, as risk to health increases with an adult weight gain of 5 kg or from a BMI of 22 kg/m^2^[32].

The majority of women reported they were actively trying to prevent weight gain over the past year, yet few were successful. This lack of success is also demonstrated by an ongoing increase in the prevalence of obesity in women in Australia and in many other countries.[33] The data here suggests many women are motivated to self-manage weight, but make largely unsuccessful changes to behavior. Interventions should be designed to enhance effectiveness of self-management strategies currently used by women and target those women who are within the healthy weight range but have begun experiencing small steady annual weight gain.

Attendance was voluntary and we may have attracted a more motivated group than is found in the population in general. As most women wished to weigh less, it is possible women participated in this lifestyle intervention with the intention of controlling their weight rather than for specific health improvements associated with diet and physical activity change. This implies body weight is a strong motivator for attendance at such programs. The weight management practices described in our study is comparable to that described in large population studies.[34] Therefore weight issues may motivate many women to seek assistance for changing behaviors, and overall this may be a stronger influence than for single component behavior change, such as changing diet composition or physical activity levels alone.

Defining the minimum level of contact and support to achieve desired outcomes in interventions is critical. Intensive programs increase the burden on participants and are costly, and low intensity mail based interventions have shown poorer outcomes but have higher population reach. In addition women who nominate a preference for group contact do not always attend as planned, [35] which has implications for delivery. In this study, attendance progressively declined over the three group sessions. We propose three face to face sessions are the limit for mothers who need to re-arrange usual activities and work commitments to attend. Non attendance may also occur because of a lack of engagement, inability of the program to meet individual expectations, or factors related to the facilitator. Retention rates were high at 4 months and women reported the sessions to be very helpful, suggesting the program quality met expectations.

Intervention strategies such as 'delivery by a health professional' and 'sessions held at school' were highly valued. Similar programs should be delivered by health professionals, a trusted source of health information, in local settings. 'Getting a walking group started at school' was considered least helpful. It was anticipated the school setting would provide strong social support for participants, and social support has been shown to be strongly correlated with physical activity in women. [36] However the unstructured format of the walking groups was not sustainable. Future research could focus on how women successfully acquire and maintain social support for physical activity.

Children have been nominated as barriers to the adoption of healthy lifestyles in women. Lack of time, lack of childcare to assist exercise opportunities and preparing meals that children prefer, have been reported elsewhere.[37] We would expect the diet of women with children will differ from childless women, although there is no comparative nutrient intake data available. Ball et al report women who live with children are less likely to meet dietary recommendations for fat intake and 'extra' foods than women living alone.[38] In our sample, the total dietary fat and saturated fat intake were found to be similar to population data, and higher than recommended by successful weight loss and chronic disease prevention interventions. The Diabetes Prevention Program aimed for a total fat intake less than 30% of energy, the Women's Healthy Lifestyle Project, less than 25% and the Women's Health Initiative, 20%.[8,39,40] A low fat intake is frequently recommended for weight management. Self reported dietary intake is often underestimated, so actual intake in this group may be even higher than reported. These results are in contrast to almost half of the participants claiming to have made changes toward a low fat diet. Overall this is pointing to a discrepancy between perceived dietary fat intake and actual fat intake and confirms prior studies. The difference may reflect a lack of knowledge on sources of fat in the diet or inaccuracies associated with self-reported intake. This has important implications for weight gain prevention strategies suggesting a role for further education on the appropriate quantity and quality of dietary fat intake in this population.

Participants were more confident in changing diet behaviors than physical activity. Women with children are likely to perceive a change in diet is within their personal control and skill level. They may also perceive fewer barriers to dietary change than for physical activity change, such as, arranging child care, re-arranging schedules, cost, weather and a lack of places to be active.

Reported average steps in healthy adults range from 7,000–13,000 per day [25] and our sample fell within that range. Reduced activity associated with having children has been reported previously [4] and step rates were expected to be lower than that observed. Much of the activity in this group appears to be low intensity presumably associated with home duties and caring for children. Women are likely to find it difficult to increase physical activity levels when already fatigued through extensive low intensity activity. Specific advice on participation in all levels of activity, low, moderate, vigorous and sitting time might improve overall activity in this group. The pedometers were sealed to eliminate feedback potentially motivating participants to be more active than usual. It is possible that simply wearing the pedometer may increase motivation to increase step counts in some women.

Limitations included recruitment of a slightly more educated sample than the general population. Education level may affect rate of weight gain.[41] Also we attracted few full time working mothers, as the intervention was held primarily during the day. Women with children are likely to find attending programs out of work hours difficult. Workplace interventions may be more appropriate for this group.

We have reached a group of women who are dissatisfied with their current weight, are attempting to control their weight, and are more confident of changing their diet than physical activity. The lifestyle behaviors exhibited by this population are not robust enough to prevent weight gain and are possibly contributing to an increased risk of cardiovascular disease. Dyslipidemia when combined with other risk factors such as overweight, low activity, and poor diet increases the risk of CVD occurring at an earlier age. The findings highlight the need for increased risk awareness and lifestyle change at earlier life stages to improve long term health outcomes in women.

Overall the results support the need for small adjustments in diet and physical activity behaviors in women which is likely to have important consequences for body weight and health. This study is novel as it delivers the intervention in a school setting aimed at the mothers. The strengths of this study are the randomized controlled design, the large representative sample of community based women and data collection using both self reported and objective methods. If successful long term, the intervention could be adapted for use in various community settings involving women, and would be a valuable addition to interventions aimed at children in a school setting.

## Conclusion

We have demonstrated that the recruitment of participants and the delivery of a low intensity lifestyle intervention to prevent weight gain in women with children is appropriate and feasible. Women with children are currently engaging in lifestyle behaviors which are not conferring adequate health benefits and may be contributing to an increased risk of cardiovascular disease. They appear to be motivated to attend prevention programs by their interest in weight management. Future research should determine the sustainability and effectiveness of low intensity behaviour change interventions focusing on preventing weight gain in this target population.

## Competing interests

The authors declare that they have no competing interests.

## Authors' contributions

CL developed, delivered and coordinated the intervention, collected and analyzed the data and wrote the manuscript. HT contributed to the intellectual design of the intervention, interpretation of data and critically revised the manuscript for important intellectual content. AD contributed to the psychosocial content and design of the intervention and questionnaires. DJ contributed to the design of the intervention and advised on the statistical analysis. All authors read and reviewed the manuscript.

## Pre-publication history

The pre-publication history for this paper can be accessed here:



## References

[B1] Australian Women and their Weight – a growing problem. http://www.alswh.org.au/Reports/achievements_reports.html.

[B2] Willett WC, Manson JE, Stampfer MJ, Colditz GA, Rosner B, Speizer FE, Hennekens CH (1995). Weight, weight change, and coronary heart disease in women. Risk within the 'normal' weight range. Journal of American Medical Association.

[B3] Williamson DF, Madans J, Pamuk E, Flegal KM, Kendrick JS, Serdula MK (1994). A prospective study of childbearing and 10-year weight gain in US white women 25 to 45 years of age. Int J Obes Relat Metab Disord.

[B4] Brown W, Trost S (2003). Life transitions and changing physical activity patterns in young women. American Journal of Preventive Medicine.

[B5] Trost SG, Sallis JF, Pate RR, Freedson PS, Taylor WC, Dowda M (2003). Evaluating a Model of Parental Influence on Youth Physical Activity. American Journal of Preventive Medicine.

[B6] Hardeman W, Griffin S, Johnston M, Kinmonth AL, Wareham NJ (2000). Interventions to prevent weight gain: a systematic review of psychological models and behaviour change methods. International Journal of Obesity.

[B7] Jeffery RW, French SA (1999). Preventing weight gain in adults: The Pound of Prevention study. American Journal of Public Health.

[B8] DPP Research Group (2002). The Diabetes Prevention Program (DPP): Description of lifestyle intervention. Diabetes Care.

[B9] Hivert MF, Langlois MF, Berard P, Cuerrier JP, Carpentier AC (2007). Prevention of weight gain in young adults through a seminar-based intervention program. International Journal of Obesity.

[B10] Levine MD, Klem ML, Kalarchian MA, Wing RR, Weissfeld L, Qin L, Marcus MD (2007). Weight Gain Prevention among Women. Obesity.

[B11] Andajani-Sutjahjo S, Ball K, Warren N, Inglis V, Crawford D (2004). Perceived personal, social and environmental barriers to weight maintenance among young women: A community survey. International Journal of Behavioral Nutrition and Physical Activity.

[B12] Shaw K, O'Rourke P, Del Mar C, Kenardy J (2005). Psychological interventions for overweight or obesity.. Cochrane Database of Systematic Reviews.

[B13] Wadden TA, Crerand CE, Brock J (2005). Behavioral treatment of obesity. Psychiatric Clinics of North America.

[B14] Foster C, Hillsdon M, Thorogood M (2005). Interventions for promoting physical activity. Cochrane Database of Systematic Reviews.

[B15] Peterson JA, Yates BC, Atwood JR, Hertzog M (2005). Effects of a Physical Activity Intervention for Women. West J Nurs Res.

[B16] Socio-Economic Indexes for Area's (SEIFA). http://www.abs.gov.au/websitedbs/d3310114.nsf/home/Census+data.

[B17] Food for Health: Dietary Guidelines for Australian Adults. http://www.health.gov.au/internet/wcms/publishing.nsf/Content/health-pubhlth-strateg-food-recommend.htm.

[B18] National Physical Activity Guidelines for Adults. http://www.health.gov.au/internet/wcms/publishing.nsf/Content/health-pubhlth-strateg-active-index.htm.

[B19] Bandura A (1986). The explanatory and predictive scope of self-efficacy theory. Journal of Social & Clinical Psychology.

[B20] Willis LH, Slentz CA, Houmard JA, Johnson JL, Duscha BD, Aiken LB, Kraus WE (2007). Minimal versus umbilical waist circumference measures as indicators of cardiovascular disease risk. Obesity.

[B21] Wang J, Thornton JC, Bari S, Williamson B, Gallagher D, Heymsfield SB, Horlick M, Kotler D, Laferrere B, Mayer L (2003). Comparisons of waist circumferences measured at 4 sites. Am J Clin Nutr.

[B22] Hallal PC, Victora CG (2004). Reliability and validity of the International Physical Activity Questionnaire (IPAQ). Med Sci Sports Exerc.

[B23] Hodge AGG (2000). Anti-Cancer Council Victoria FFQ: Relative validity of nutrient intake compared to diet diaries in young to mid aged women in a study of iron supplementation. ANZ J Public Health.

[B24] International Physical Activity Questionnaire. http://www.ipaq.ki.se.

[B25] Tudor-Locke CE, Myers AM (2001). Methodological considerations for researchers and practitioners using pedometers to measure physical (ambulatory) activity. Res Q Exerc Sport.

[B26] Tudor-Locke C, Burkett L, Reis JP, Ainsworth BE, Macera CA, Wilson DK (2005). How many days of pedometer monitoring predict weekly physical activity in adults?. Preventive Medicine.

[B27] Sidman CL, Corbin CB, Le Masurier G (2004). Promoting physical activity among sedentary women using pedometers. Res Q Exerc Sport.

[B28] Sallis JF (1988). The development of self efficacy scales for health-related diet and exercise behaviors. Health Education Research.

[B29] Marcus BR (1992). Self efficacy and the stages of exercise behavior change. Res Q Exerc Sport.

[B30] Kristal A, Glanz K, Curry S, Patterson R (1999). How Can Stages of Change be Best Used in Dietary Interventions?. Journal of the American Dietetic Association.

[B31] Saelens BE, Gehrman CA, Sallis JF, Calfas KJ, Sarkin JA, Caparosa S (2000). Use of self-management strategies in a 2-year cognitive-behavioral intervention to promote physical activity. Behavior Therapy.

[B32] McLaren L, Kuh D (2004). Body dissatisfaction in midlife women. Journal of Women & Aging.

[B33] The Australian Diabetes Obesity and Lifestyle Study. http://www.diabetes.com.au/pdf/AUSDIAB_Report_Final.pdf.

[B34] Timperio A, Cameron-Smith D, Burns C, Crawford D (2000). The public's response to the obesity epidemic in Australia: weight concerns and weight control practices of men and women. Public Health Nutr.

[B35] Klem ML, Viteri JE, Wing RR (2000). Primary prevention of weight gain for women aged 25–34: the acceptability of treatment formats. International Journal of Obesity.

[B36] Trost S, Owen N, Bauman A, Sallis J, Brown W (2002). Correlates of adults participation in physical activity: review and update. Medicine and science in sports and exercise.

[B37] Ball K, Crawford D, Salmon J, Giles-Corti B, Mishra GD (2005). the 'SEESAW' study.

[B38] Ball K, Mishra GD, Thane CW, Hodge A (2004). How well do Australian women comply with dietary guidelines?. Public Health Nutr.

[B39] Simkin-Silverman LR, Wing RR, Boraz MA, Kuller LH (2003). Lifestyle intervention can prevent weight gain during menopause: results from a 5-year randomized clinical trial. Annals of Behavioral Medicine.

[B40] Howard BV, Manson JE, Stefanick ML, Beresford SA, Frank G, Jones B, Rodabough RJ, Snetselaar L, Thomson C, Tinker L (2006). Low-Fat Dietary Pattern and Weight Change Over 7 Years: The Women's Health Initiative Dietary Modification Trial. JAMA.

[B41] Burke GL, Bild DE, Hilner JE, Folsom AR, Wagenknecht LE, Sidney S (1996). Differences in weight gain in relation to race, gender, age and education in young adults: the CARDIA study. Ethn Health.

